# DNA methylation in diabetic retinopathy: pathogenetic role and potential therapeutic targets

**DOI:** 10.1186/s13578-022-00927-y

**Published:** 2022-11-17

**Authors:** Chunyang Cai, Chunren Meng, Shuai He, Chufeng Gu, Thashi Lhamo, Deji Draga, Dawei Luo, Qinghua Qiu

**Affiliations:** 1 Department of Ophthalmology, Shanghai General Hospital, Shanghai Jiao Tong University School of Medicine, Shanghai, People’s Republic of China; 2 National Clinical Research Center for Eye Diseases, Shanghai Key Laboratory of Ocular Fundus Diseases, Shanghai Engineering Center for Visual Science and Photomedicine, Shanghai Engineering Center for Precise Diagnosis and Treatment of Eye Diseases, Shanghai, People’s Republic of China; 3 Department of Ophthalmology, Shigatse People’s Hospital, Shigatse, Xizang People’s Republic of China

**Keywords:** Diabetic retinopathy, DNA methylation, Epigenetics, Pathogenic mechanisms, Therapeutic targets

## Abstract

**Background:**

Diabetic retinopathy (DR), a specific neuron-vascular complication of diabetes, is a major cause of vision loss among middle-aged people worldwide, and the number of DR patients will increase with the increasing incidence of diabetes. At present, it is limited in difficult detection in the early stages, limited treatment and unsatisfactory treatment effects in the advanced stages.

**Main body:**

The pathogenesis of DR is complicated and involves epigenetic modifications, oxidative stress, inflammation and neovascularization. These factors influence each other and jointly promote the development of DR. DNA methylation is the most studied epigenetic modification, which has been a key role in the regulation of gene expression and the occurrence and development of DR. Thus, this review investigates the relationship between DNA methylation and other complex pathological processes in the development of DR. From the perspective of DNA methylation, this review provides basic insights into potential biomarkers for diagnosis, preventable risk factors, and novel targets for treatment.

**Conclusion:**

DNA methylation plays an indispensable role in DR and may serve as a prospective biomarker of this blinding disease in its relatively early stages. In combination with inhibitors of DNA methyltransferases can be a potential approach to delay or even prevent patients from getting advanced stages of DR.

## Introduction

Diabetic retinopathy (DR) is an insidious progressive neuron-vascular complication of diabetes mellitus (DM). This common complication is a major reason for irreversible vision impairment in middle-aged adults (20–65 years) [1]. It is estimated that approximately 642 million people worldwide will have DM by 2040 [2], and one-third of them will suffer from symptoms induced by DR [3]. At first, patients are asymptomatic, but when their vision starts to deteriorate, the DR may already be in its advanced stage [4]. The early symptoms are microaneurysms and intraretinal hemorrhage. As the disease progresses, the number and size of the hemorrhages greatly increase, obstructed precapillary arterioles damage the nerve fiber layer, and cotton-wool spots begin to occur [5]. Finally, the capillaries are not perfused, and new blood vessels appear, resulting in proliferative diabetic retinopathy (PDR), which can lead to serious complications such as retinal detachment and even blindness [6]. However, there are limited clinical methods for the early diagnosis [7] and treatment [4, 8] of DR. Thus, it is very important to study effective diagnostic methods and new therapeutic targets for early onset of this blinding disease.

Epigenetics is regarded as a phenomenon that is beyond genetics [9], in which epigenetic modifications can activate or silence genes [10]. The classical definition is that epigenetics are stable and heritable changes in gene function caused by alterations in chromosomes without changes in the DNA sequence [11, 12]. Different from inherited genetic changes, which are static over the course of life, epigenetic modifications are dynamic and influenced by the environment, lifestyle and disease [6]. However, once changes occur, they may remain stable for a period of time [13]. Three primary types of epigenetic mechanisms are DNA methylation, histone modifications, and noncoding RNA expression [14]. Different modification mechanisms independently regulate specific epigenetic phenomena but can interact with each other and regulate several physiological processes together [15]. Among these, DNA methylation acts as an intermediary between external factors and the genome [16] and is involved in important pathophysiological processes, including embryonic development, stem cell differentiation, tumorigenesis and aging [17].

Accumulated studies have demonstrated that epigenetic modification plays a critical role in different pathogenic mechanisms of DR, including oxidative stress [18], inflammation [19], and neovascularization [20]. In addition, persistent status of epigenetic modification becomes a major driving force of metabolic memory [21]. A positive relationship between DNA methylation and DR development has been discovered, suggesting that a high DNA methylation status may be a potential risk factor for DR [15]. Therefore, this review mainly focuses on how DNA methylation contributes to the development of DR via these various pathogenic mechanisms. In addition, from this new perspective, we can explore potential molecular biomarkers for relatively early diagnosis and prognosis and novel measures to prevent and treat DR.

## Dynamic process of DNA methylation

DNA methyltransferases (Dnmts) methylate DNA at the fifth carbon of cytosines by transferring methyl groups from S-adenosyl methionine (SAM), thus forming 5-methylcytosine (5mC) [22]. In mammals, DNA methylation predominantly occurs at CpG dinucleotides in the human genome [23], resulting in gene repression [14]. When CpG is methylated, certain transcription factors are unable to bind to the DNA sequence, thus affecting transcription [18]. In addition, methylated CpG binding proteins (MeCP) recruit inhibitors to the promoter region where methylation occurs, resulting in gene transcription silencing [24]. In the diabetic environment, Dnmts are activated, and the expression of Dnmt1 is increased in the retina and its vascular system [16].

**Fig. 1:**
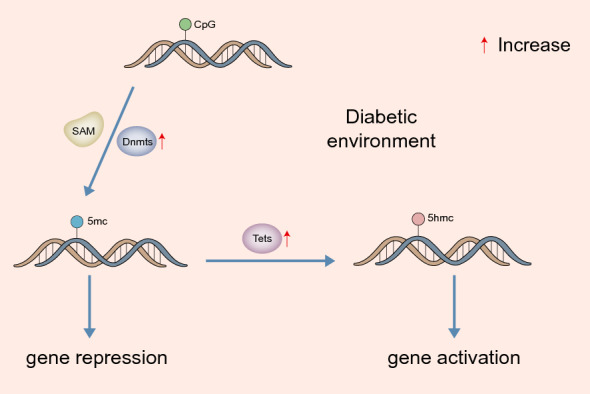
Dynamic process of DNA methylation. In the diabetic environment, Dnmts and Tets are activated. Dnmts act at the 5′-position of cytosine residues in CpG islands to form 5mC and contribute to gene repression. Tets can quickly hydroxymethylate 5mC to 5hmC and promote gene activation. Dnmts, DNA methyltransferases; Tets, ten-eleven translocation dioxygenases; SAM, S-adenosyl methionine; 5mC, 5-methylcytosine; 5hmC, 5-hydroxymethylcytosine

Ten-eleven translocation dioxygenases (Tets) are common demethylation enzymes, which can quickly hydroxymethylate 5mC to 5-hydroxymethylcytosine (5hmC) [25]. This opens up the chromatin for the binding of the transcription factor, and activation and expression of specific genes are stimulated [26]. Tets are also activated in the retina and its vascular system in diabetic patients, and Tet2 is the most closely related and significant subtype in the pathogenesis of DR and has been widely studied [18, 27] (Fig. 1).

## The role of DNA methylation in the pathogenesis of DR

DNA methylation is closely related to normal and pathological development of the human retina. On the one hand, in normal cells, it ensures the accuracy of gene expression patterns in time and space and is necessary for mammalian development [28]. For example, DNA methylation has been proven to maintain specific gene expression patterns that vary by cell type in retinal cells, such as photoreceptors and nonphotoreceptor cells [29]. On the other hand, aberrant DNA methylation is related to retinal diseases, including age-related macular degeneration (AMD) [30], DR [31] and retinitis pigmentosa [32].

Here, we further investigated how aberrant DNA methylation contributes to the pathological process of DR via three other pathways, including oxidative stress, inflammation, and neovascularization. In addition, there is an interesting phenomenon in DR, called metabolic memory.

### DNA methylation in oxidative stress of DR

#### Oxidative stress and DR

Oxidative stress is a general term that mainly describes toxic effects to cells, tissues, or organs caused by reactive oxygen species (ROS) [33]. Excessive ROS can damage cell structures, including lipids, membranes, proteins, and nucleotides [34]. The retina is a suitable target for oxidative damage, as retinal cells, including rod and cone photoreceptors, have high metabolic rates and oxygen consumption rates [35]. It has been shown that increased cytosolic ROS induced by diabetes is an early process in the retinal vascular system, which occurs earlier than mitochondrial damage and histopathological development [36]. ROS can impair mitochondria and accelerate capillary cell apoptosis in the retina, eventually resulting in DR [37].

#### DNA methylation and oxidative stress

**Fig. 2:**
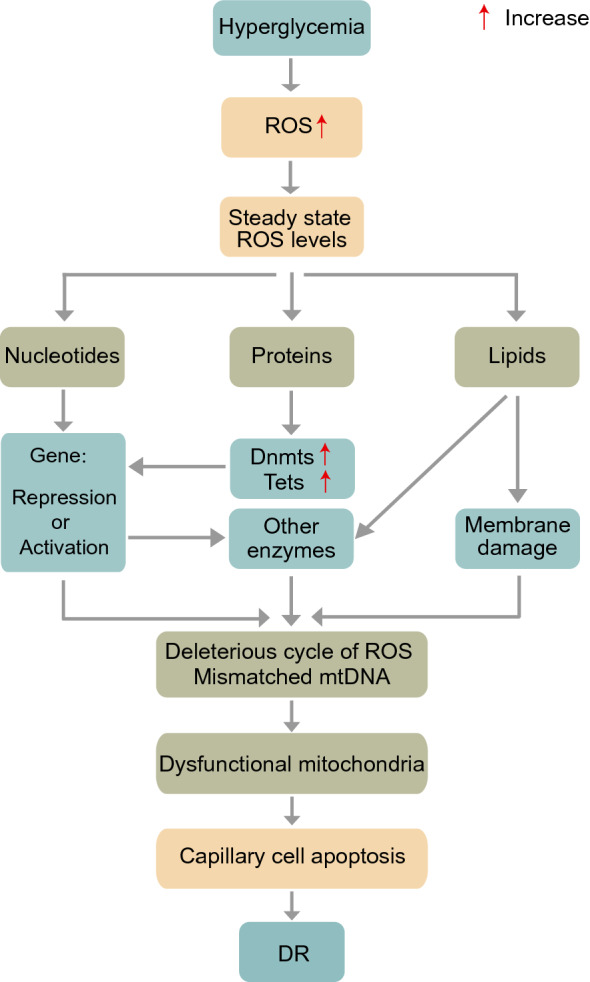
Mechanisms for oxidative stress leading to dysfunctional mitochondria in the retina. Increased ROS levels compromise nucleotides, proteins and lipids and alter gene expression, enzyme activity and membrane function. Together, these changes lead to a deleterious cycle of ROS and mismatched mtDNA. Ultimately, DR development is promoted. ROS, reactive oxygen species; Dnmts, DNA methyltransferases; Tets, ten-eleven translocation dioxygenases; mtDNA, mitochondrial DNA; DR, diabetic retinopathy

During the pathological process of DR, oxidative stress affects DNA methylation. On one hand, oxidative stress influences DNA methylation. ROS are the active intermediates of DNA methylation and can participate in epigenetic processes by nucleophilic substitution reactions [38]. Dnmts, the enzymes that modulate methylation status, are sensitive to redox reactions [39]. ROS production is able to activate these enzymes, promoting DNA methylation by deprotonating cytosine molecules [38]. The function of Dnmts can be regulated by oxidative stress through a potential dual effect. The early effects inhibit the activity of Dnmts and produce the highest levels of ROS, while the long-term effects improve the activity and expression of Dnmts [40]. On the other hand, DNA methylation promotes oxidative stress and ultimately contributes to the development of DR [41]. The possible mechanisms are explained by abnormal DNA methylation in the following related genes (Fig. 2).

##### mtDNA-related methylation: POLG1, D-loop, and MLH1

**Fig. 3:**
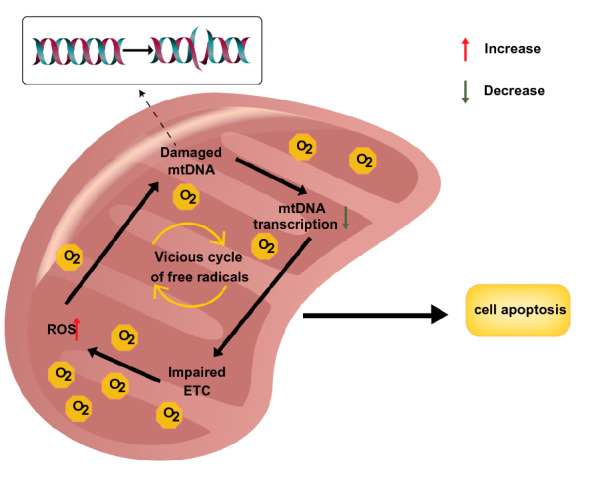
A deleterious cycle of ROS in the mitochondria in the diabetic environment. The continuous increase in ROS in the cytoplasm impairs mtDNA, as well as transcription and the ETC. These changes further exacerbate mitochondrial dysfunction, promote a vicious cycle of ROS and initiate the process of cell apoptosis. ROS, reactive oxygen species; mtDNA, mitochondrial DNA; ETC, electron transport chain

Due to damage from DM, the retinal mitochondria become swollen when their membrane potential is damaged, leading to the elevation of superoxide radicals and impairment of respiration [6]. In addition, a compromised electron transport chain (ETC) system influences the efficient transfer of electrons during oxidative phosphorylation and gives rise to ROS, leading to a positive feedback loop in which ROS promote oxidative stress and consequently generate more ROS [42]. Finally, excessive cytochrome c from dysfunctional mitochondria leaks into the cytoplasm, initiating the process of apoptosis [43] (Fig. 3).

DNA methylation, common in nuclear DNA (nDNA), is also observed in mitochondrial DNA (mtDNA), which is equipped with Dnmts and Tets [44]. In diabetes, Dnmts expression is activated, and mtDNA is hypermethylated in retinal mitochondria, which impairs the transcription of genes encoded by mtDNA, contributes to dysfunctional mitochondria and accelerates capillary cell apoptosis [45]. Mitochondrial homeostasis is maintained through a connection between nDNA and mtDNA, where changes in nuclear gene expression resulting from aberrant DNA methylation may compromise mitochondrial functionality [22]. Polymerase gamma 1 (POLG1) is a catalytic subunit of the nuclear-encoded enzyme, which is involved in mtDNA replication [46]. In addition to binding POLG1 with the displacement loop (D-loop) to regulate its replication, POLG is involved in repairing mtDNA damage [47]. Due to retinal Dnmts, which is activated in DM, the regulatory site of POLG1 is hypermethylated [45]. This hypermethylation impairs mtDNA biogenesis and its transcription and damages the ETC system, speeding up the deleterious loop of ROS [21]. The D-loop region is a critical control point for mtDNA transcription and replication [48]. Compared with other regions of mtDNA in DM, D-loop is more highly methylated and disrupts the transcription of mtDNA-encoded genes that are indispensable for maintaining the ETC system, which increases the electron leakage of the ETC complexes. These harmful results directly produce excessive superoxide radicals, aggravating the development of DR [45]. In addition, the D-loop is the site where mtDNA binds to the inner mitochondrial membrane and is more vulnerable to damage than other regions of mtDNA [49].

During the development of DR, retinal mitochondria are dysfunctional, and mtDNA is impaired due to an increase in base mismatches and hypermethylation of cytosines [50]. In addition, the status of mtDNA methylation is positively correlated with increased base mismatches [50]. MutL homolog 1 (MLH1) is an enzyme important in the DNA mismatch repair (MMR) pathway [51]. Hyperglycemia conditions reduce MLH1 mitochondrial localization and hypermethylate its promoter with activated Dnmt1 [52]. Finally, the repression of gene transcripts leads to mtDNA mismatches and mitochondrial damage. In summary, the studies mentioned above suggest that the balanced state of DNA methylation may play a role in preventing mtDNA damage and slowing down or inhibiting the progression of retinal pathology.

##### Matrix metalloproteinase-9 (MMP-9)

MMP-9 is an enzyme implicated in retinal mitochondrial damage, and its transcription is mediated by its DNA methylation status [53]. In retinal endothelial cells, hyperglycemia increases the binding of Dnmt1 and Tet2 to the promoter of the MMP-9 gene [16]. After Dnmt1 adds a methyl group to the cytosine to form 5mC, Tet2 demethylates that cytosine into 5hmC, activating MMP-9 transcription [54]. Finally, increased activity and transcription of the MMP-9 gene can compromise retinal mitochondria and promote oxidative stress, leading to the development of DR [41].

##### Ras-related C3 botulinum toxin substrate 1 (RAC1)

NADPH oxidases (Noxs) generate the majority of cytosolic ROS in retinal cells, which is an early process during the development of DR, and RAC1 is an obligatory component of Nox2 [55]. In the retina of DM, RAC1 is activated both functionally and transcriptionally, which is related to the DNA methylation conditions of its promoter [18]. When Dnmts and Tets are both activated in DM [16], although Dnmt1 forms 5mC at the RAC1 promoter, a concomitant increase in Tet2 hydroxymethylates it to 5hmC, activating the transcription of RAC1 [18]. This result promotes Nox2-ROS-mitochondrial damage and fuels capillary cell apoptosis, consequently leading to DR [56].

##### Peroxisome proliferator-activated receptor alpha (PPARα)

As a transcription factor, PPARα participates in the regulation of oxidative stress [57]. In human retinal capillary pericytes (HRCPs) that are treated with high glucose, Dnmt1 is highly expressed and interacts with the PPARα promoter, enhancing its DNA methylation levels and repressing PPARα expression. Consequently, the number of apoptotic cells and ROS levels are dramatically elevated [17]. In addition, a recent study reveals that Dnmts inhibition can inhibit PPARα methylation and reduce the destruction of retinal cells [17]. These results provide strong evidence that Dnmt1-mediated DNA methylation of PPARα accelerates apoptosis and ROS, increasing dysfunctional retinal cells. However, whether the methylation status of PPARα can affect inflammation in DR is still unclear.

##### Mitochondrial superoxide dismutase (MnSOD)

MnSOD is responsible for scavenging mitochondrial superoxide and preventing mitochondrial dysfunction [58]. In DM, decreased MnSOD levels may lead to increased ROS levels and play a critical role in pericyte loss of DR [59]. Studies have demonstrated that DNA methylation of the MnSOD promoter in the DR group is higher than that in the NO-DR group and that MnSOD transcription in the DR group is significantly lower than that in the NO-DR group [49]. This indicates that the retina in diabetes experiences hypermethylation of a specific promoter and decreased MnSOD activity, which eventually develops into DR.

### DNA methylation in the inflammation of DR

#### Inflammation and DR

**Fig. 4:**
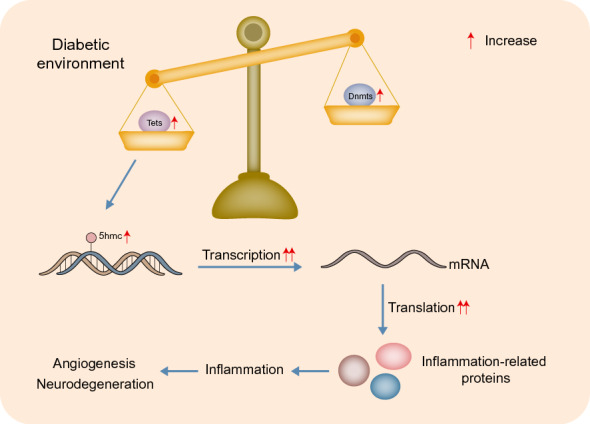
Process for aberrant DNA methylation of key genes inducing an inflammatory response in the retina. Diabetes contributes to hypomethylation of the promoter in inflammation-related genes. Finally, increased transcription and translation of inflammatory cytokines result in angiogenesis and neurodegeneration in retinopathy. Dnmts, DNA methyltransferases; Tets, ten-eleven translocation dioxygenases; 5hmC, 5-hydroxymethylcytosine

Inflammation is a nonspecific defense against injury or stress [60]. Transcription of functional mediators, including pro-inflammatory cytokines, acute phase proteins and chemokines, is elevated, which act in the recruitment and activation of monocytes and leukocytes and in the subsequent inflammatory process [61]. DR is considered a chronic inflammatory disease related to the immune inflammatory response, which contributes to retinal angiogenesis and neurodegeneration [62] (Fig. 4). Retinal microvascular disease, an early pathological process in DR, is caused by low-level, persistent leukocyte activation, recurrent capillary obstruction and progressive, destructive retinal ischemia [63]. Local retinal inflammation is regulated by activated microglia, which exist in the retinal plexiform layers when long-term tissue stress induces microglia to overreact and change into a phenotype that secretes proinflammatory mediators [64]. Studies have found various inflammation-related phenomena in the retina of DR patients, including leukostasis, neutrophil and macrophage infiltration, complement and microglial activation, and increased vascular permeability [61]. Moreover, the inhibition of proinflammatory molecules has been proven to prevent pathological processes in rats with DR [65].

#### DNA methylation and inflammation

Studies have shown that an abnormal DNA methylation status is closely related to intraocular inflammation [19, 66]. The aberrant DNA methylation of some key genes that induce the inflammatory response in the retina of DM patients is described below.

Tumor necrosis factor (TNF), a proinflammatory cytokine, is increased in serum, and the DNA methylation of its promoter decreases in cases with PDR [67]. These findings provide evidence that DNA methylation of the TNF gene in DM patients can disrupt the normal expression of TNF, resulting in inflammation in DR.

NOD-like receptor (NLRP3) is important in the formation and activation of inflammatory bodies [68] and is associated with transforming growth factor beta-1 (TGFβ1) [69], monocyte chemoattractant protein-1 (MCP-1) [70], and tumor necrosis factor ligand superfamily member 2 (TNFSF2) [71], which together play a role in the development of vascular complications in DM. Studies have found that DR patients display reduced DNA methylation levels of NLRP3, suggesting the aberrant expression of inflammatory corpuscles in DR [62]. Moreover, compared with healthy individuals, levels of DNA methylation in the TGFβ1, MCP-1 and TNFSF2 genes are also significantly reduced in DR patients [62]. These findings prove that hypomethylation in the promoter of these inflammatory response-related genes participates in DR development.

Metastasis-associated lung adenocarcinoma transcript 1 (MALAT1), a prominent intergenic long noncoding RNA (lncRNA), is well known for its role in influencing epigenetic mechanisms in cancer [72]. In DM, the inhibition of DNA methylation in the CpG island of its promoter elevates the expression of inflammatory factors in human retinal endothelial cells [17]. MALAT1 may regulate the inflammatory response through independent and dependent pathways [73]. together contributing to the development of DR through a complex inflammatory response.

### Neovascularization

#### Neovascularization and PDR

When DR progresses, capillary perfusion may be impaired, leading to retinal ischemia, followed by the upregulation of proangiogenic factors, thus leading to pathologic neovascularization. PDR is the progressive phase of DR, of which a typical feature is the pathological proliferation of new vessels [74]. In patients with DM, the excitation of complex cascade signals increases angiogenic factors and mediates the proliferation of retinal endothelial cells, promoting aberrant neovascularization [75]. Vascular endothelial growth factor (VEGF), a potent angiogenic factor, is secreted by both endothelial and nonendothelial cells [74], and angiogenesis induced by VEGF is an important compensatory response to retinal microvascular sparsity in DM, leading to the occurrence of PDR [76].

#### DNA methylation and neovascularization

Hypomethylated CpG in the promoter region plays a role in increased expression of the VEGF gene [77]. This indicates that DNA methylation participates in the process of neovascularization and promotes the occurrence and development of PDR. Maternally expressed gene 3 (MEG3) is a lncRNA that is related to cellular proliferation and apoptosis [78]. It has a blocking effect on the development of DR, as its overexpression can decrease the pathological expression of VEGF [79] and suppress the endothelial-mesenchymal transition [80]. However, its transcription is dramatically reduced in DR [81]. Studies have demonstrated that Dnmt1 promotes DNA methylation of the MEG3 promoter to impede MEG3 expression [80], accelerating proliferation, migration, and neovascularization in human retinal microvascular endothelial cells [82]. Moreover, one study found that CpG methylation can regulate angiogenesis pathways in DR progression, such as ETS1, HES5, and PRDM16 [20].

### Metabolic memory

#### Metabolic memory and DR

Diabetes-related complications can occur or continue to develop in diabetic patients with prolonged hyperglycemia, even if the blood glucose is controlled to normal levels with proper treatment [83]. This phenomenon is known as “metabolic memory” [84]. Surprisingly, even short-term hyperglycemia has been proven to result in this metabolic memory [85]. The “metabolic memory” phenomenon also exists in DR. Even after the hyperglycemic insult has terminated, the deleterious effects of hyperglycemia on retinal tissues continue and depend on the duration and severity of this insult [86]. In addition, it was found that 25.1% of type 2 diabetes (T2D) patients with good glycemic control had DR, which was associated with their HbA1c levels over the previous 5 years and their skin autofluorescence (SAF) levels [87].

#### DNA methylation and metabolic memory

Persistent epigenetic changes induced by hyperglycemia are a major driving force underlying metabolic memory [88, 89]. Studies have found that in retinal cells exposed to hyperglycemia, both the activity and expression of Dnmts are increased [40], and that the mtDNA replication system is impaired because of continued hypermethylation of POLG [47]. These changes continue even after the hyperglycemic injury has ceased [47], suggesting that the DNA methylation mechanism in retinal and capillary cells continues to be activated and that the DNA status in nDNA and mtDNA continues to change [21]. Because of DNA hypermethylation, the transcription of genes, including those critical to mitochondrial homeostasis, remains impaired, and dysfunctional mitochondria continue to accelerate the development of DR [21, 90].

During an 18-year follow-up, DNA methylation of some specific “persistent” CpGs has been shown to mediate the relationship between a history of hyperglycemia and the development of future complications (retinopathy) [91]. Surprisingly, these variations in DNA methylation can alter enhancer activity in stem cells, myeloid cells, and other cells [92]. This indicates that metabolic memory in humans may be due to the establishment of epigenetic alterations in stem cells induced by hyperglycemia [93]. Even after removing the initial stimulus, these alterations persist in differentiated cells for a long time and lead to chronic diabetic complications that are difficult to treat with conventional therapies [91]. However, more research is needed to substantiate this theory and further explore other possible mechanisms of metabolic memory.

### Aberrant DNA methylation of other key genes

#### Methylenetetrahydrofolate reductase (MTHFR)

MTHFR is an enzyme that accelerates the synthesis of methyl radicals for the homocysteine (Hcy) cycle, providing methyl groups for DNA methylation [94, 95]. Studies have shown that a high methylation status in the promoter of the MTHFR gene contributes to variations in MTHFR expression, leading to dysregulation of Hcy metabolism [96]. The elevation of Hcy in plasma levels results in increased oxidative stress, which induces increased proinflammatory cytokines and a dysfunctional endothelium [96], significantly increasing the progression of DR [97]. Furthermore, it has been demonstrated that the hypermethylation status of the MTHFR gene is closely related to high levels of total cholesterol, low-density lipoprotein (LDL) and DR in patients with T2D [98, 99]. These studies show that altered methylation of the MTHFR gene promoter is involved in the development of DR and influences both Hcy and lipid metabolism.

#### Sirtuin 1 (SIRT1)

SIRT1, a histone deacetylase, regulates gene transcription and multiple biological processes, including cell proliferation and apoptosis [100]. Overexpression of SIRT1 can not only prevent an increase in capillary cell apoptosis and the occurrence of degenerative capillaries but also protect the overall health of the retinal vascular system [101]. These findings prove that it has a protective effect on the progression of DR. Due to high DNA methylation of the SIRT1 promoter in DM [102], its transcription is inhibited [103], accompanied by deteriorative apoptosis, capillary degeneration and low vascular density [101]. It is closely related to the development of DR and damage to retinal blood vessels and neurons [6, 104]. In addition, SIRT1 can in turn balance the DNA methylation status of its promoter when SIRT1 reduces Dnmt1 activation at the Dnmt1 promoter, and decreased Dnmt1 reduces DNA methylation of the SIRT1 promoter, thus regulating its transcription [101].

## Diagnosis and prognosis of DR: DNA methylation-related biomarkers

### Diagnosis of DR at a relatively early stage

DR is an insidious progressing chronic disease, while the gap between the diagnosis of DM and the discovery of any sign of DR can be as long as 10 or even 20 years [49]. Clinical manifestations of vascular abnormalities in the retina that occur long after the occurrence of DR are the main basis for its clinical diagnosis [5, 105]. This makes the discovery of effective biomarkers critical. Levels of global DNA methylation are regulated in the early stages of diabetes or possibly before [106] and have been proven to be higher in patients with DR than in diabetic patients without retinopathy [17]. Therefore, it may act to predict patients at risk of developing DM or DR [107]. Moreover, as mentioned before, DNA methylation at mtDNA and the promoter of critical enzymes is strongly correlated with the occurrence of DR. One study showed that mtDNA methylation in peripheral blood differs between diabetic patients with retinopathy and those without retinopathy [49]. In addition, it has been demonstrated that a low methylation status of the NLRP3, TGFβ1, MCP-1 and TNFSF2 promoters may elevate the risk of DR [62], suggesting that these genes may be used as possible targets for detecting DR.

As the most frequently assayed epigenetic mark, DNA methylation can be easily and quickly quantified by methylation-specific PCR [108]. In addition, its changes can be detected noninvasively in inflammatory cells such as blood monocytes and lymphocytes [88]. More importantly, DNA methylation can precede disease pathology [15]. Thus, DNA methylation status in the peripheral blood of DM patients can be used as a promising biomarker [49], which enables a relatively earlier diagnosis than existing clinical measures and a prognostic prediction for this blinding disease [32, 109].

These findings open up the possibility of using abnormal DNA methylation in peripheral blood as a noninvasive biomarker to diagnose DR earlier than existing clinical methods. However, whether there is a sufficient aberrant methylation status in the plasma of patients in the early stage for detection and what its specificity and sensitivity are need to be further studied [110]. This indicates that whether it can be used as a diagnostic marker for the early stage and even for the occurrence of DR remains to be explored by more basic and clinical studies.

### Prediction of the progressive stage of DR (PDR)

DNA methylation status may possibly be used as a noninvasive biomarker for predicting the progressive stage of DR, known as PDR [49]. It has been demonstrated that the DNA methylation of some genes in peripheral blood can be used as a prospective biomarker of PDR in patients with type 1 diabetes. These genes include the following: (1) TNF; (2) chitinase 3-like protein 1 (CHI3L1), which participates in tissue injury, inflammation, tissue repair, and remodeling responses;[111] (3) chimerin 2 (CHN2), which may be a key element of proximal insulin signaling, playing a role in insulin resistance and growth deficiency;[112] and (4) gastric inhibitory polypeptide receptor (GIPR), which encodes glucose-dependent insulinotropic polypeptide receptor [113]. Moreover, these genes show reduced DNA methylation in PDR patients [67]. In addition, it has been reported that some specific genes involved in the cytotoxicity pathway mediated by natural killer cells are hypomethylated in PDR patients [67], further suggesting that different methylation patterns can serve as promising markers of PDR.

### Prediction of the effectiveness of drug therapy

As mentioned above, DNA methylation of some important CpG islands may help in the diagnosis of this blinding disease. Thus, it is possible to use the methylation status of these genes as novel biomarkers to predict the efficacy of specific drugs. Markers based on DNA methylation in liquid biopsies are regarded as important prognostic factors for four cancer types, including lung, breast, colorectal and prostate cancers [108]. In addition, it has been demonstrated that the methylation status of the promoter of VEGF receptor genes can potentially be used to predict the efficacy of drugs that target VEGF in cancer cells [114]. Unfortunately, this kind of research in DR is not sufficient at present, but further research on the key genes mentioned previously is expected to provide a possibility for predicting the effectiveness of DR therapies by using DNAme-related markers.

## Role of DNA methylation in the prevention and treatment of DR

As mentioned before, in DR, an aberrant DNA methylation status is not the same for different genes. Hypermethylation of some genes contributes to DR, while hypomethylation of others leads to DR. In summary, when the dynamic methylation balance of these key genes is disrupted, the occurrence and development of DR is promoted. Next, this review will explore the role of DNA methylation in the prevention and treatment of DR.

### Prevention via risk factors for DR

Risk factors for DR, including hyperglycemia, obesity, dyslipidemia, hyperhomocysteinemia (HHcy), lifestyle and environment, may alter the epigenetic status of different target tissues independently or synergistically [88]. Here, we focus on these factors, mainly through the control of the daily diet, to avoid abnormal DNA methylation and prevent the occurrence and development of DR.

#### Hyperglycemia

As mentioned before, hyperglycemia serves as the starting point of aberrant DNA methylation in diabetes [93, 115], and therefore glycemic control is a key preventable measure for reducing the risk of DR and vision loss [116]. First, in the initial stages of DM, reestablishment of good glycemic control prevents retinal mitochondria from being compromised when the activation of Dnmts and Tets and the methylation of mtDNA and nDNA remain unchanged [21]. This shows that by controlling blood glucose early and maintaining it at good levels, the DNA methylation mechanism does not damage the retina of diabetic patients. Additionally, even if patients cannot or do not have strict glycemic control in the initial phase of DM, long-term strict glycemic control can still improve abnormal methylation status and ultimately delay or stop the development of DR [21]. Therefore, from the aspect of DNA methylation, it has been confirmed again that good blood glucose control is important for preventing and delaying the development of DR. In addition, regardless of the stage of diabetes, it is indispensable for DM patients to control blood glucose.

#### Hyperhomocysteinemia (HHcy)

HHcy is an independent risk factor for the development of retinopathy [117] and plays an important role in retinal microvascular damage [118]. In addition, it may produce synergistic detrimental effects with hyperglycemia [119]. Under physiological conditions, Hcy can form SAM and increase Dnmts activity to maintain normal DNA methylation status [120]. However, in DR, increased circulating Hcy induces a global increase in retinal DNA methylation [120]. Further effects on the methylation of genes critical for mtDNA biogenesis induce a deleterious cycle of ROS and mitochondrial dysfunction, contributing to the development of DR [121]. Therefore, we can apply all kinds of measures that can control the level of circulating Hcy to prevent DR. For example, folic acid and vitamin B12 are important in maintaining normal Hcy metabolism, and HHcy could be the result of their deficiency [122]. Therefore, treating pre-existing HHcy with folic acid and vitamin B12 may help to reduce the risk of DR [123].

#### Hyperlipidemia/obesity

Some cross-sectional and longitudinal studies indicate an association between an increased risk of DR and hyperlipidemia/obesity [124, 125]. In DM rats, hyperlipidemia/obesity exaggerates hyperglycemia-induced DNA methylation, increases Dnmts and Tet2, and alters methylation of mtDNA and RAC1. Consequently, mitochondrial damage and retinopathy are accelerated [126]. In addition, increased consumption of sugar and processed food is one of the risk factors for a dramatically increased incidence of T2D and plays an important role in the retinal dysfunction of overweight DR patients [6, 127]. Thus, it is recommended that DM patients need to maintain proper BMI and levels of lipids through proper drugs, physical activity and less consumption of sugar and processed food [127], which could be beneficial for avoiding aberrant DNA methylation and preventing DR.

### Treatment: targeting DNA methylation

#### Targeting enzymes associated with DNA methylation: Dnmt inhibitors

There are three main types of Dnmts that exert biological activities: Dnmt1, Dnmt3a and Dnmt3b. Dnmt1 can regulates tissue-specific patterns of methylated cytosine residues [6]. Targeting Dnmts to inhibit DNA methylation has been used in many chronic diseases. For example, the US Food and Drug Administration has already approved the Dnmt inhibitors 5-azacytidine and 5-aza-20-deoxycytidine for myeloid cancers and cutaneous T-cell lymphoma [6]. Research on Dnmt inhibitors has evolved from non-selective to selective. First-generation Dnmt inhibitors inhibited all three Dnmts (1, 3a and 3b), including 5-azacitidine and 5-aza-2′-deoxycytidine, whereas recent studies found that nanaomycin A and GSK3685032 selectively inhibited Dnmt3b and Dnmt1, respectively [128].

Dnmt inhibitors may play a potential role in DR treatment [129]. As previously stated, DR progression does not stop after termination of hyperglycemia, where the DNA methylation-hydroxymethylation mechanism in the retina cannot be improved immediately with glycemic control [86]. However, direct inhibition of Dnmts during this reversal phase could improve aberrant DNA methylation, which alleviates persistent mitochondrial dysfunction and delays/stops the development of DR [21]. For example, direct inhibition of Dnmts can restore DNA methylation and gene transcription of the MLH1 gene during periods of good glycemic control following reversal of hyperglycemia [130]. In addition, DNA methylation may be one of the mechanisms that disrupts antioxidant defense systems during DR development [41]. Dnmt inhibitors can prevent mtDNA damage by preventing further DNA methylation of genes that are important in maintaining mitochondrial homeostasis if they are supplemented before the acceleration of apoptosis in retinal capillary cells [130]. A DNA-demethylating drug known as 5-aza-2′-deoxycytidine can reverse increased Dnmts and regulate the expression of antioxidant enzymes to improve antioxidative capacity, indicating its promising effect in treating DR [129, 131]. Although 5-aza-2′-deoxycytidine is non-selective and has inhibitory effects on three Dnmts, it has not been found to have significant damage to retinal structure [130, 131], which may be related to the general increase in activity of all Dnmts in the diabetic environment. However, there is relatively few research in this area, and selective inhibitors can be further investigated to improve efficacy and reduce side effects. For example, Dnmt1 has tissue-specific [6] and irreplaceable [132] characteristics. By further studying the distribution characteristics of Dnmt1 in retinal tissue, the feasibility of Dnmt1 selective inhibitor in the treatment of DR can be explored.

#### Targeting methyl donors for DNA methylation: folic acid and vitamin B12

Folic acid (FA) is a dietary methyl donor for DNA methylation [133], while vitamin B12 is a cofactor involved in the process of transferring the methyl group from FA to DNA [134]. FA has been widely used in the clinic for DR treatment, while studies suggest that low FA levels can result in both hypomethylation and hypermethylation, leading to misregulation of DNA methylation [27, 135]. Low levels of folic acid/vitamin B12 in serum increase the risk of DR [117, 136], and their supplementation contributes to a decreased risk of DR [27]. This suggests that deficiencies in FA and vitamin B12 may promote the development of DR by influencing the homeostasis of DNA methylation, further indicating the role of FA and vitamin B12 in DR treatment. However, the DNA methylation-related mechanism underlying its therapeutic effect needs to be further elucidated.

#### Synergistic therapy with current therapies

Current treatment strategies for DR aim to control microvascular complications, including intravitreal drugs, laser photocoagulation, and vitreous surgery [82]. Among them, intravitreal injection of an anti-VEGF antibody and dexamethasone implants are first-line therapies, but these therapies have several limitations [5]. Based on the current studies, our view is that Dnmts inhibitors have the potential to be a complementary therapy for current therapies. Through this synergistic therapy, the adverse effects and limitations of conventional therapy can be decreased, and the therapeutic effect for DR can be improved [114].

Anti-VEGF therapy is mainly used in PDR patients, and has a significant effect on neovascularization and diabetic macular edema, which can improve visual acuity [137]. The main problems are as follows: (1) a large part of the population is not sensitive to anti-VEGF therapy [138]. Studies have shown that the DNA methylation status of VEGF receptor promoter can affect the therapeutic effect of VEGF-targeted drugs [114]. Therefore, the DR patients who are not sensitive to anti-VEGF therapy may be associated with the abnormal DNA methylation of VEGF receptor promoter caused by diabetes. Anti-VEGF therapy in combination with Dnmt inhibitors may be a potential approach to improve the therapeutic effects of those special DR patients. (2) Anti-VEGF therapy is mainly used in the PDR stage with obvious ocular symptoms, and Dnmt inhibitors are expected to prevent entering the PDR stage. As mentioned previously, DNA methylation in peripheral blood is expected as a diagnostic biomarkers of early DR. With close follow-up before the diagnosis of PDR, Dnmt inhibitors can be used to reverse epigenetic abnormalities caused by hyperglucemia in a short period of time. Combined with the good control of blood glucose in the later stage, it is very promising to delay or even prevent DR from developing into PDR. (3) Considering the chronic course of DR and the short-term efficacy of anti-VEGF therapy, PDR patients should be regularly and frequently injected with anti-VEGF for a period of time [138]. The disruption of this treatment can be devastating, possibly leading to irreversible vision loss [138]. However, the inconvenience of follow-up and heavy economic burden make it difficult for many patients to achieve good compliance [5, 139]. Dnmt inhibitors may be used as complementary therapies to improve patient compliance by prolongation of action time and reduction of cost.

Dexamethasone implants are currently available in long-acting products that can be effective for up to 16 weeks. The main problems are as follows: (1) dexamethasone and other cortisol substances are hormones, which cause obvious side effects and high incidence of adverse reactions for long-term intraocular application, such as increased intraocular pressure, induced glaucoma, and induced/accelerated cataract. It is contraindicated in patients with periocular infections, glaucoma, and implant allergies [140]. (2) The main effect of such cortisol implants is to regulate inflammation response, but its role in PDR remains unclear [5]. Abnormal DNA methylation is involved in the activation of many inflammatory factors and pathways in DR. Therefore, Dnmt inhibitors have the potential to be used as a complementary therapy to regulate inflammation response during the PDR phase or as an alternative option for patients with contraindications to cortisol implant devices.

## Conclusion and prospects

**Fig. 5:**
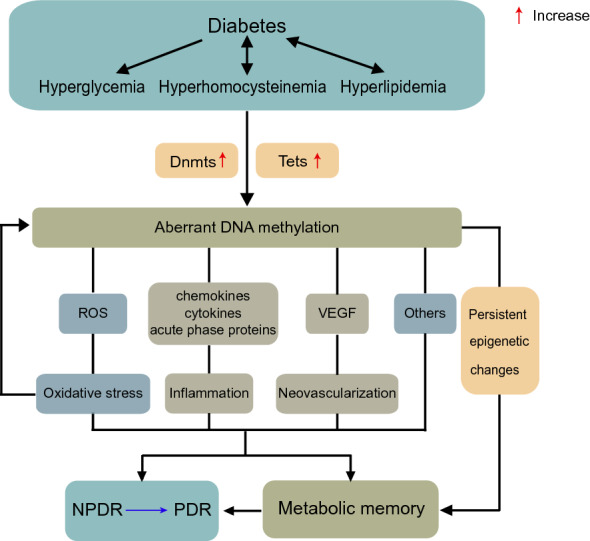
Abnormal DNA methylation contributes to DR through various pathogenic mechanisms. Diabetes is accompanied by metabolic disorders, including hyperglycemia, hyperhomocysteinemia, and hyperlipidemia. Dnmts and Tets are activated in the diabetic environment, and subsequent aberrant DNA methylation contributes to DR via oxidative stress, inflammation, and neovascularization. In addition, aberrant DNA methylation can be promoted by oxidative stress in turn, and its persistent status becomes a major driving force of metabolic memory. Dnmts, DNA methyltransferases; Tets, ten-eleven translocation dioxygenases; ROS, reactive oxygen species; VEGF, vascular endothelial growth factor; NPDR, non-proliferative diabetic retinopathy; PDR, proliferative diabetic retinopathy

In conclusion, DNA methylation plays an indispensable role in the occurrence and development of DR via complex pathological processes, which includes oxidative stress, inflammation, neovascularization and metabolic memory (Fig. 5, Table 1). It may become a potential biomarker for early diagnosis and a prospective target for the prevention and treatment of DR. However, to date, therapies for DR are relatively limited, mainly for the neovascularization of patients with PDR, while studies on DNA methylation are still in their infancy. Dnmt inhibitors are promising therapies to treat DR in the future. However, considering that most of these inhibitors currently under study are nonselective, it will be important to identify which type of Dnmt inhibitor regulates the antioxidant genes and the exact hypermethylation CpG site of the genes. When these inhibitors are used for treatment of DR, are there side effects that are associated with cancer treatment, such as myelosuppression and neurological toxicity? Do these inhibitors significantly change the structure and function of the retina? Do diabetic patients develop tolerance to acute inhibition of retinal Dnmts? Do Dnmts inhibitors can make up for the deficiency of anti-VEGF drugs and cortisol implant devices? All of these questions need to be taken into consideration for future research. Thus, further research on DNA methylation is expected to bring new breakthroughs in the treatment of DR and new hope to patients who are blind due to DR.

**Table 1:** List of DR-related genes and their aberrant methylation status

Pathogenesis	Key genes	DNA methylation status	References
Oxidative stress	PLOG1	Hypermethylation	Mishra et al. [45]
	D-loop	Hypermethylation	Mishra et al. [45]
	MLH1	Hypermethylation	Mohammad et al. [52]
	MMP-9	Hypomethylation	Zhou et al. [54]
	RAC1	Hypomethylation	Duraisamy et al. [18]
	PPARα	Hypermethylation	Zhu et al. [17]
	MnSOD/SOD2	Hypermethylation	Duraisamy et al. [49]
Inflammation	NLRP3, TGFβ1, MCP-1, TNFSF2	Hypomethylation	Chen et al. [62]
	MALAT1	Hypomethylation	Zhu et al. [17]
Neovascularization	MEG3	Hypermethylation	He et al. [80]
	ETS1, HES5, PRDM16	Unknown	Berdasco et al. [20]
Others	MTHFR	Hypermethylation	Dos Santos Nunes et al. [96]
			Santana Bezerra et al. [98]
	SIRT1	Hypermethylation	Mishra et al. [101]
			Chen et al. [102]

POLG1, polymerase gamma 1; D-loop, displacement loop; MLH1, MutL homolog 1; MMP-9, matrix metalloproteinase-9; RAC1, Ras-related C3 botulinum toxin substrate 1; PPARα, peroxisome proliferator-activated receptor alpha; MnSOD, mitochondrial superoxide dismutase; NLRP3, NOD-like receptor 3; TGFβ1, transforming growth factor beta-1; MCP-1, monocyte chemoattractant protein-1; TNFSF2, tumor necrosis factor ligand superfamily member 2; MALAT1, metastasis-associated lung adenocarcinoma transcript 1; MEG3, maternally expressed gene 3; MTHFR, methylenetetrahydrofolate reductase; SIRT1, sirtuin 1

## Data Availability

Not applicable.

## Abbreviations

DR: Diabetic retinopathy
DM: Diabetes mellitus
PDR: Proliferative diabetic retinopathy
5mC: 5-Methylcytosine
Dnmts: DNA methyltransferases
SAM: S-adenosyl methionine
MeCP: Methylated CpG binding proteins
5-hmC: 5-Hydroxymethylcytosine
Tets: Ten-eleven translocation dioxygenases
AMD: Age-related macular degeneration
ROS: Reactive oxygen species
ETC: Electron transport chain
nDNA: Nuclear DNA
mtDNA: Mitochondrial DNA
POLG1: Polymerase gamma 1
D-loop: Displacement loop
MLH1: MutL homolog 1
MMP-9: Matrix metalloproteinase-9
RAC1: Ras-related C3 botulinum toxin substrate 1
Noxs: NADPH oxidases
PPARα: Peroxisome proliferator-activated receptor alpha
HRCPs: Human retinal capillary pericytes
MnSOD: Mitochondrial superoxide dismutase
TNF: Tumor necrosis factor
NLRP3: NOD-like receptor
TGFβ1: Transforming growth factor beta-1
MCP-1: Monocyte chemoattractant protein-1
TNFSF2: Tumor necrosis factor ligand superfamily member 2
MALAT1: Metastasis-associated lung adenocarcinoma transcript 1
lncRNA: Long noncoding RNA
VEGF: Vascular endothelial growth factor
MEG3: Maternally expressed gene 3
T2D: Type 2 diabetes
SAF: Skin autofluorescence
MTHFR: Methylenetetrahydrofolate reductase
Hcy: Homocysteine
LDL: Low density lipoprotein
SIRT1: Sirtuin 1
CHI3L1: Chitinase 3-like protein 1
CHN2: Chimerin 2
GIPR: Gastric inhibitory polypeptide receptor
HHcy: Hyperhomocysteinaemia
